# Role of renin–angiotensin system antagonists on long-term mortality post-percutaneous coronary intervention in reduced and preserved ejection fraction

**DOI:** 10.1007/s00392-021-01985-x

**Published:** 2022-01-20

**Authors:** Hamish C. Prosser, Kah Yong Peck, Diem Dinh, Louise Roberts, Jaya Chandrasekhar, Angela Brennan, Stephen J. Duffy, David Clark, Andrew E. Ajani, Ernesto Oqueli, Martin Sebastian, Christopher M. Reid, Melanie Freeman, Jithin K. Sajeev, Andrew W. Teh

**Affiliations:** 1 Department of Cardiology, Eastern Health, Box Hill Hospital, Level 2, 8 Arnold Street, Box Hill, VIC 3128 Australia; 2 Eastern Health Clinical School, Monash University, Melbourne, VIC Australia; 3 Department of Epidemiology and Preventive Medicine, Monash University, Melbourne, VIC Australia; 4 Department of Cardiology, Alfred Health, The Alfred Hospital, Melbourne, VIC Australia; 5 Department of Cardiology, Austin Hospital Clinical School, The University of Melbourne, Melbourne, VIC Australia; 6 Department of Cardiology, Royal Melbourne Hospital, Parkville, VIC Australia; 7 Department of Cardiology, Ballarat Health Services, Ballarat, VIC Australia; 8 Department of Cardiology, Barwon Health, University Hospital, Geelong, VIC Australia; 9 School of Public Health, Curtin University, Perth, WA Australia; 10 School of Medicine, Deakin University, Geelong, Australia

**Keywords:** Angiotensin converting enzyme inhibitors, Angiotensin receptor blockers, STEMI/NSTEMI, Percutaneous coronary intervention, Heart failure

## Abstract

**Aims:**

The use of angiotensin-converting enzyme inhibitors (ACEi) or angiotensin II-receptor blockers (ARBs) post-myocardial infarction (MI) is supported by evidence based on trials performed in the thrombolysis era. This was prior to primary percutaneous coronary intervention (PCI) being routine practice, and with little direct evidence for the use of these medications in patients with preserved left ventricular (LV) function. This study sought to determine whether there is an association between ACEi/ARB use after PCI for acute coronary syndrome (ACS) and long-term all-cause mortality, with a particular focus on patients with preserved LV function.

**Methods:**

This multicentre, observational study evaluated prospectively collected data of 21,388 patients (> 18 years old) that underwent PCI for NSTEMI and STEMI between 2005 and 2018, and were alive at 30 day follow-up.

**Results:**

In total, 83.8% of patients were using ACEi/ARBs. Kaplan–Meier analysis demonstrated ACEi/ARB use was associated with a significantly lower mortality in the entire cohort (15.0 vs. 22.7%; *p* < 0.001) with a mean follow-up of 5.58 years; and independently associated with 24% lower mortality by Cox proportional hazards modelling (HR 0.76, CI 0.67–0.85, *p* < 0.001). ACEi/ARB therapy was also associated with significantly lower mortality in patients with reduced or preserved LV function, with greater survival benefit with worse LV dysfunction.

**Conclusion:**

ACEi/ARB therapy post-PCI is associated with significantly lower long-term mortality in patients with reduced and preserved LV function. These findings provide contemporary evidence for using these agents in the current era of routine primary PCI, including those with preserved EF.

**Graphical abstract:**

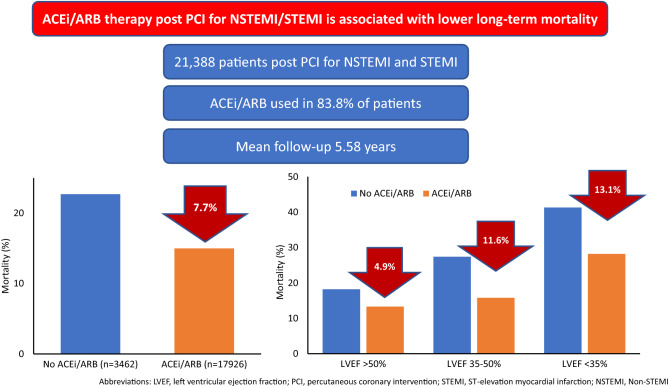

**Supplementary Information:**

The online version contains supplementary material available at 10.1007/s00392-021-01985-x.

## Introduction

Optimal medical therapy plays a critical role in preventing further cardiovascular events and improving clinical outcomes following percutaneous coronary intervention (PCI) for ACS [1–3]. As such, international guidelines recommend multiple medications for secondary-prevention including anti-platelet agents, a β-adrenergic receptor blocker (β-blocker), and a statin [4–7]. Angiotensin-converting enzyme inhibitors (ACEi) or angiotensin receptor blockers (ARBs) are also recommended as first-line therapy in patients with concomitant heart failure (HF), left ventricular (LV) dysfunction (ejection fraction (EF) ≤ 40%), anterior MI, diabetes or hypertension, and in those with stable chronic kidney disease (CKD) [4–8]. Guidelines also recommend that ACEi may be considered for all patients regardless of these associated factors, but this is supported by a reduced level of evidence (Class IIa, level A-B evidence) [4–8].

The evidence underlying guideline recommendations comes from large trials mostly performed over 20 years ago, during an era prior to routine and/or primary PCI, where thrombolysis was often performed, with greater subsequent mortality compared to the present time. The last 20 years have seen major changes in the management of ACS, resulting in a greater proportion of patients with preserved LV function, primarily due to increased use of primary PCI, and a routine early invasive strategy in NSTEMI [2, 9].

The aims of this study were (1) identify prescribing practices of RAS inhibitors (ACEi and ARBs) in patients post-PCI for MI between 2005 and 2018; (2) evaluate the long-term survival benefits of ACEi/ARBs in an unselected cohort of patients that underwent PCI for STEMI or NSTEMI; (3) determine the effects of ACEi/ARB therapy in patients with reduced and preserved LV function by subgroup analysis.

## Methods

### Study design and patient population

This multicenter, observational study utilized prospectively collected data from the Melbourne Interventional Group (MIG) registry, an Australian registry that collects comprehensive data on consecutive patients undergoing PCI from six major public (government funded) hospitals within Victoria, Australia [10]. Data from a total of 21,388 patients that underwent PCI for STEMI and NSTEMI between January 2005 and September 2018 were evaluated. Inclusion criteria required the patient to be > 18 years old, undergo PCI for NSTEMI or STEMI, and be contactable at 30-day follow up where their use of ACEi/ARB and clinical outcomes were recorded. Exclusion criteria included patients that died prior to, or were not contactable at 30-day follow up, and those in which use of ACEi/ARB was not able to be determined (discharge medications are not recorded in the MIG registry). Long-term mortality was identified via linkage to the Australian National Death Index, with multiple patient demographics cross-matched with the MIG registry to identify deceased patients. The ethics committee of each participating hospital had approved registry participation. Consent was obtained in all participants via an “opt-out” model. This study abided by the ethical guidelines of the 1975 Declaration of Helsinki.

### Outcomes

The primary outcome of this study was long-term, all-cause mortality. The cohort was stratified by patient LV ejection fraction (EF) into < 35%, 35–50% and > 50%. LVEF was measured or estimated within 4 weeks of patient admission, the vast majority during the inpatient stay.

### Statistical analysis

Continuous variables are expressed as mean ± standard deviation, with categorical variables expressed as numbers and percentages as indicated. Differences between groups in discrete variables relating to patient characteristics and clinical outcomes were analyzed by Pearson Chi-squared tests. All data were tested for normal distribution prior to selecting the appropriate statistical test. Where appropriate, independent sample *t* tests (for normally distributed data) or Kruskal–Wallis equality-of-populations rank tests (for non-normally distributed data) were used for continuous variables. Survival curves were determined by Kaplan–Meier analysis, with differences in survival assessed by log-rank tests. A *p* value of < 0.05 was considered to indicate statistical significance. Differences in baseline characteristics which may have acted as potential confounders to the analysis were accounted for using Cox proportional multivariable modelling, as previously published [11]. Variables found to have a *p* value < 0.10 in the univariable model were considered for inclusion in the multivariable model to identify whether they remained statistically significant and independent predictors of mortality. The full list of variables included in this modelling is included in Supplementary Table 1. Statistical analyses were performed using Stata software (Stata 16.0, College Station, TX, USA).

## Results

### Baseline patient characteristics

**Fig. 1:**
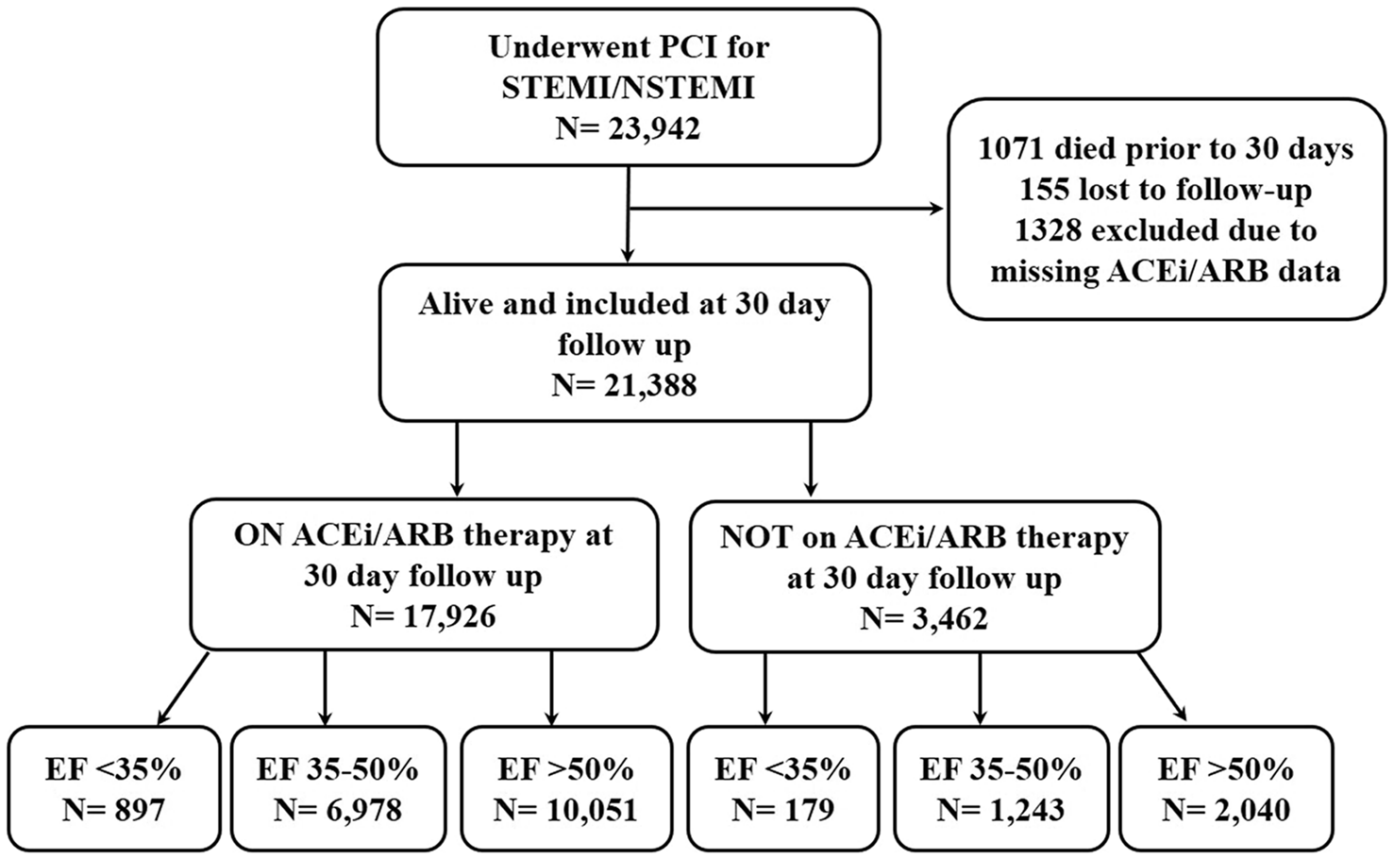
Flow diagram illustrating the study population and subgroups. *ACEi* angiotensin converting enzyme inhibitor, *ARB* angiotensin receptor blocker, *EF* left ventricular ejection fraction, *NSTEMI* non-ST-segment elevation myocardial infarct, *STEMI* ST-segment elevation myocardial infarct

Between 2005 and 2018 23,942 consecutive patients underwent PCI for STEMI or NSTEMI. Of these 2554 were excluded and the remaining 21,388 patients alive at 30 day follow-up were evaluated (Fig. 1). At 30 days, 17,926 patients (83.8%) were taking an ACEi/ARB, while 3462 (16.2%) were not (Table 1). At baseline, those who were on ACEi/ARB therapy at 30 days were younger, hypertensive, with elevated BMI, and a family history of CAD. The group not prescribed ACEi/ARB had significantly more women and comorbidities including chronic lung disease, prior MI, prior CABG, prior HF, prior valvular surgery, peripheral vascular disease, cerebrovascular disease, renal disease requiring dialysis, and rheumatoid arthritis (Table 1).

**Table 1:** Patient baseline characteristics

	All patients			LVEF < 35%			LVEF 35–50%			LVEF > 50%		
	ACEi/ARBs *n* = 17,926	No ACEi/ARBs *n* = 3462	*p* value	ACEi/ARBs *n* = 897	No ACEi/ARBs *n* = 179	*p* value	ACEi/ARBs *n* = 6978	No ACEi/ARBs *n* = 1243	*p* value	ACEi/ARBs *n* = 10,051	No ACEi/ARBs *n* = 2040	*p* value
Age (years)	63.5 ± 12.3	64.6 ± 13.2	< 0.001	65.6 ± 13.0	66.9 ± 13.7	0.23	63.5 ± 12.4	65.9 ± 13.6	< 0.001	63.2 ± 12.1	63.6 ± 12.8	0.233
Male sex (%)	77.5%	74.2%	< 0.001	75.7%	72.1%	0.34	79.5%	75.1%	< 0.001	76.4%	73.9%	0.018
BMI (kg/m^2^)	28.6 ± 5.4	27.4 ± 5.4	< 0.001	27.4 ± 5.2	26.1 ± 5.5	0.004	28.3 ± 5.2	27.2 ± 5.3	< 0.001	28.8 ± 5.5	27.6 ± 5.4	< 0.001
Diabetes (%)	21.0%	21.7%	0.434	29.5%	30.2%	0.87	21.1%	22.3%	0.32	20.3%	20.5%	0.837
NIDDM (%)	15.8%	13.5%		21.2%	16.8%		15.8%	13.1%		15.5%	13.4%	
IDDM (%)	5.2%	8.2%	< 0.001	8.3%	13.4%	0.06	5.3%	9.2%	< 0.001	4.8%	7.1%	< 0.001
Smoking status												
Current (%)	33.0%	32.6%		33.0%	22.3%		33.8%	32.1%		32.5%	33.7%	
Previous (%)	34.2%	33.5%		33.8%	32.5%		33.4%	32.7%		34.7%	34.1%	
Never smoked (%)	32.8%	33.9%	0.449	33.2%	45.2%	0.004	32.8%	35.3%	0.223	32.8%	32.2%	0.571
Chronic lung disease (%)	9.9%	12.4%	< 0.001	12.5%	12.4%	0.98	9.6%	12.2%	0.005	9.9%	12.5%	< 0.001
Hypertension (%)	60.8%	52.2%	< 0.001	60.9%	69.3%	0.03	58.6%	52.6%	< 0.001	62.3%	50.5%	< 0.001
Dyslipidemia (%)	57.8%	56.4%	0.109	56.4%	53.6%	0.50	55.0%	53.0%	0.195	59.9%	58.6%	0.277
Prior MI (%)	17.3%	19.6%	0.001	29.3%	26.8%	0.50	17.7%	20.5%	0.020	15.9%	18.5%	0.005
Prior PCI (%)	15.1%	16.0%	0.194	20.9%	11.2%	0.003	14.7%	15.9%	0.269	14.9%	16.5%	0.074
Prior CABG (%)	5.0%	5.8%	0.038	9.5%	8.4%	0.65	5.0%	6.4%	0.030	4.6%	5.3%	0.215
Prior valvular surgery (%)	0.5%	1.2%	< 0.001	0.6%	5.0%	< 0.001	0.5%	1.1%	0.010	0.5%	0.9%	0.033
Family history of CAD (%)	37.5%	34.8%	0.003	30.0%	19.5%	0.01	36.7%	31.6%	0.001	38.8%	37.9%	0.473
PVD (%)	4.6%	6.7%	< 0.001	9.0%	10.6%	0.51	5.1%	7.1%	0.004	3.8%	6.1%	< 0.001
Prior HF (%)	2.7%	3.8%	< 0.001	14.2%	9.5%	0.09	2.7%	5.2%	< 0.001	1.6%	2.6%	0.002
Cerebrovascular Disease (%)	4.5%	6.5%	< 0.001	6.0%	9.5%	0.09	4.9%	6.7%	0.007	4.2%	6.1%	< 0.001
OSA (%)	3.7%	4.1%	0.259	3.1%	2.8%	0.82	3.5%	3.6%	0.994	3.9%	4.6%	0.148
Rheumatoid arthritis (%)	1.9%	2.7%	0.003	1.4%	2.9%	0.17	2.1%	3.7%	0.001	1.7%	2.0%	0.450
Baseline creatinine	91.5 ± 57.2	117.6 ± 126.9	< 0.001	106.1 ± 83.2	150.9 ± 131.8	< 0.001	93.4 ± 65.3	122.7 ± 128.8	< 0.001	89.0 ± 47.8	111.7 ± 124.9	< 0.001
eGFR > 60 (%)	79.6%	70.0%		64.6%	41.9%		79.6%	66.9%		80.9%	74.2%	
eGFR 30 to < 60 (%)	18.6%	21.8%		31.1%	39.0%		18.2%	23.6%		17.7%	19.4%	
eGFR < 30 (%)	1.8%	8.2%	< 0.001	4.3%	19.1%	< 0.001	2.2%	9.5%	< 0.001	1.4%	6.4%	< 0.001
Dialysis (%)	0.7%	4.2%	< 0.001	1.5%	6.2%	< 0.001	0.9%	5.2%	< 0.001	0.6%	3.5%	< 0.001

*ACEi* angiotensin converting enzyme inhibitor, *ARB* angiotensin II receptor blocker, *BMI* body mass index (kg/m^2^), *CABG* coronary artery bypass graft, *CAD* coronary artery disease, *eGFR* estimated glomerular filtration rate (mL/min/1.73 m^2^), *HF* heart failure, *IDDM* insulin-dependent diabetes mellitus, *LVEF* left ventricular ejection fraction, *MI* myocardial infarction, *NIDDM* non-IDDM, *OSA* obstructive sleep apnea, *PCI* percutaneous coronary intervention, *PVD* peripheral vascular disease

These baseline differences between treatment groups from the entire cohort were generally mirrored in the subgroups EF 35–50% (*N* = 8221) and EF > 50% (*N* = 12,091). Few patient characteristics differed between treatment groups in the smaller cohort of patients with EF < 35% (*N* = 1076), however, significantly fewer were on ACEi/ARB if hypertensive or a current smoker, and a greater proportion were on ACEi/ARB in the setting of prior PCI.

### Prescribing practices of ACEi/ARBs at 30-day follow-up

**Fig. 2:**
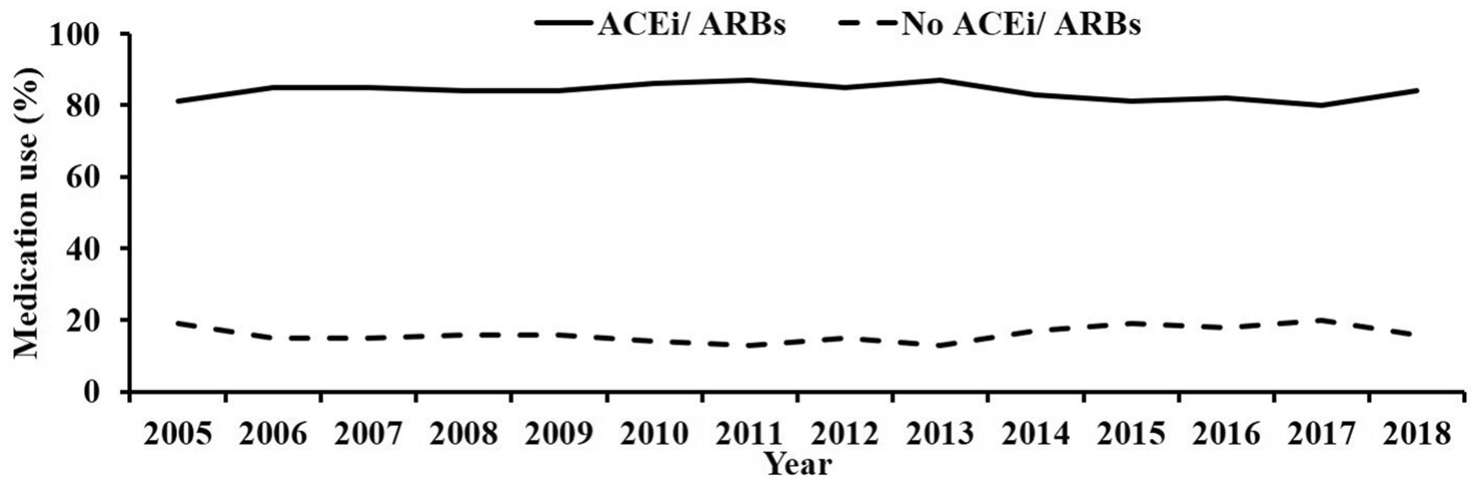
ACEi/ARB use across the study period 2005–2018. The total rate of ACEi/ARB use is 83.8% (*p* < 0.001 versus no ACEi/ARB use). The trend over time shows a small but significant decline in ACEi/ARB use (*p* = 0.001). *ACEi* angiotensin converting enzyme inhibitor, *ARB* angiotensin receptor blocker

Observing the use of ACEi/ARBs in all patients post-PCI between 2005 and 2018, the proportion of patients receiving ACEi/ARB each year fluctuated mildly between 80 and 87% with a small but statistically significant reduction in ACEi/ARB use over the study period (*p* = 0.001), and remained significantly greater than patients not using ACEi/ARB (*p* < 0.001; Fig. 2). Of the 83.8% of patients prescribed an ACEi/ARB in this study, 82.8% were prescribed ACEi and 18.2% an ARB (Table 2).

**Table 2:** Medications at 30-day follow-up

	All patients			LVEF < 35%			LVEF 35–50%			LVEF > 50%		
	ACEi/ARBs *n* = 17,926 (%)	No ACEi/ARBs *n* = 3462 (%)	*p* value	ACEi/ARBs *n* = 897 (%)	No ACEi/ARBs *n* = 179 (%)	*p* value	ACEi/ARBs *n* = 6978 (%)	No ACEi/ARBs *n* = 1243 (%)	*p* value	ACEi/ARBs n = 10,051 (%)	No ACEi/ARBs *n* = 2040 (%)	*p* value
Aspirin	97.3	95.0	< 0.001	93.1	88.8	0.05	96.8	93.6	< 0.001	98.1	96.3	< 0.001
P2Y12 inhibitors	97.2	93.5	< 0.001	93.5	89.9	0.08	96.5	91.9	< 0.001	97.9	94.9	< 0.001
Warfarin	7.4	6.0	0.003	27.3	21.8	0.13	10.7	7.7	0.001	3.4	3.6	0.632
NOAC	3.0	4.0	0.016	5.2	4.1	0.61	3.4	5.2	0.012	2.6	3.3	0.178
Nitrates	7.2	9.1	< 0.001	7.7	9.2	0.53	6.3	9.3	< 0.001	7.8	9.0	0.080
Spironolactone	2.6	3.4	0.10	15.2	12.8	0.44	3.1	4.3	0.042	1.2	2.1	0.003
Eplerenone	3.3	1.3	< 0.001	19.2	6.1	< 0.001	5.1	2.0	< 0.001	0.6	0.4	0.172
Fibrate	1.4	1.5	0.563	1.5	1.2	0.76	1.7	1.9	0.762	1.1	1.4	0.445
Ezetimibe	3.9	4.6	0.049	5.4	7.3	0.34	3.5	4.4	0.153	4.0	4.5	0.288
Statin	96.8	92.3	< 0.001	94.1	84.3	< 0.001	97.2	92.3	< 0.001	96.7	93.0	< 0.001
ACE inhibitor	82.8	0.0	< 0.001	85.9	0	< 0.001	84.8	0	< 0.001	81.4	0	< 0.001
ARB	18.2	0.0	< 0.001	16.0	0	< 0.001	16.4	0	< 0.001	19.6	0	< 0.001
β-Blocker	85.8	77.6	< 0.001	89.9	84.4	0.03	88.1	78.7	< 0.001	83.8	76.3	< 0.001
CCB	10.8	12.4	0.008	7.7	4.9	0.2	8.2	8.9	0.455	12.9	15.2	0.007

*ACE* angiotensin converting enzyme, *ARB* angiotensin II-receptor blocker, *CCB* calcium channel blocker, *LVEF* left ventricular ejection fraction, *NOAC* non-vitamin K antagonist oral anticoagulants

There were some statistically significant differences in medication regimens identified between those that were and were not on ACEi/ARB therapy (Table 2). Patients on ACEi/ARB had a significantly higher proportion also on aspirin, P2Y12 inhibitors, warfarin, eplerenone, β-blocker and statin therapy. Those not prescribed ACEi/ARB were more frequently prescribed calcium channel blockers, ezetimibe, non-vitamin K oral anticoagulants (NOAC) and nitrate therapy. The use of spironolactone did not differ between those on ACEi/ARB therapy or not. The greater prescribing of aspirin, statin, and β-blockers to those on ACEi/ARB was consistent across all LVEF subgroups (Table 2).

### Cardiac status and angiography/PCI characteristics

All patients underwent PCI, with 87% of STEMI patients on ACEi/ARB therapy at 30 days compared to 80% for NSTEMI (*p* < 0.001; Table 3). A significantly greater proportion of patients were on ACEi/ARB therapy if they had an out of hospital cardiac arrest, underwent thrombolysis, or had a successful PCI (Table 3).

**Table 3:** Cardiac status at PCI with angiography/PCI characteristics

	All patients			LVEF < 35%			LVEF 35–50%			LVEF > 50%		
	ACEi/ARBs *n* = 17,926	No ACEi/ARBs *n* = 3462	*p* value	ACEi/ARBs *n* = 897	No ACEi/ARBs *n* = 179	*p* value	ACEi/ARBs *n* = 6978	No ACEi/ARBs *n* = 1243	*p* value	ACEi/ ARBs *N* = 10,051	No ACEi/ARBs *n* = 2040	*p* value
STEMI	53.6%	41.1%		68.3%	57.5%		66.9%	55.8%		43.0%	30.7%	
NSTEMI	46.4%	58.9%	< 0.001	31.7%	42.5%	0.005	33.1%	44.2%	< 0.001	57.0%	69.3%	< 0.001
HF < 2 weeks prior (%)	4.2%	5.5%	0.001	21.8%	22.4%	0.86	5.3%	7.7%	0.001	1.8%	2.7%	0.012
Cardiogenic shock (%)	3.3%	3.5%	0.66	13.7%	20.7%	0.02	4.2%	3.8%	0.482	1.8%	1.8%	0.984
Out of hospital cardiac arrest (%)	3.7%	2.4%	< 0.001	8.5%	4.5%	0.06	4.9%	3.0%	0.003	2.4%	1.9%	0.156
Multivessel disease (%)	56.9%	56.3%	0.521	65.6%	76.0%	0.007	58.4%	59.3%	0.54	55.0%	52.7%	0.053
Lesion result successful (%)	96.7%	94.4%	< 0.001	94.2%	90.8%	0.09	96.3%	94.0%	< 0.001	97.2%	94.9%	< 0.001
Procedural medications (%)												
Gp2b3a	40.8%	34.0%	< 0.001	50.5%	39.7%	0.008	49.0%	42.1%	< 0.001	34.3%	28.5%	< 0.001
Thrombolysis	7.5%	5.3%	< 0.001	8.6%	6.1%	0.56	9.6%	8.4%	0.283	6.8%	3.4%	< 0.001
Aspirin	99.0%	98.7%	0.065	99.0%	99.4%	0.57	99.0%	98.2%	0.015	99.0%	98.9%	0.528
P2Y12 inhibitors	72.0%	72.7%	0.403	65.9%	77.1%	0.003	72.1%	72.1%	0.978	72.4%	72.7%	0.838

*ACE* angiotensin converting enzyme, *ARB* angiotensin II-receptor blocker, *HF* heart failure, *LVEF* left ventricular ejection fraction, *NSTEMI* non-ST-segment elevation myocardial infarction, *STEMI* ST-segment elevation myocardial infarction

### Clinical outcomes

**Fig. 3:**
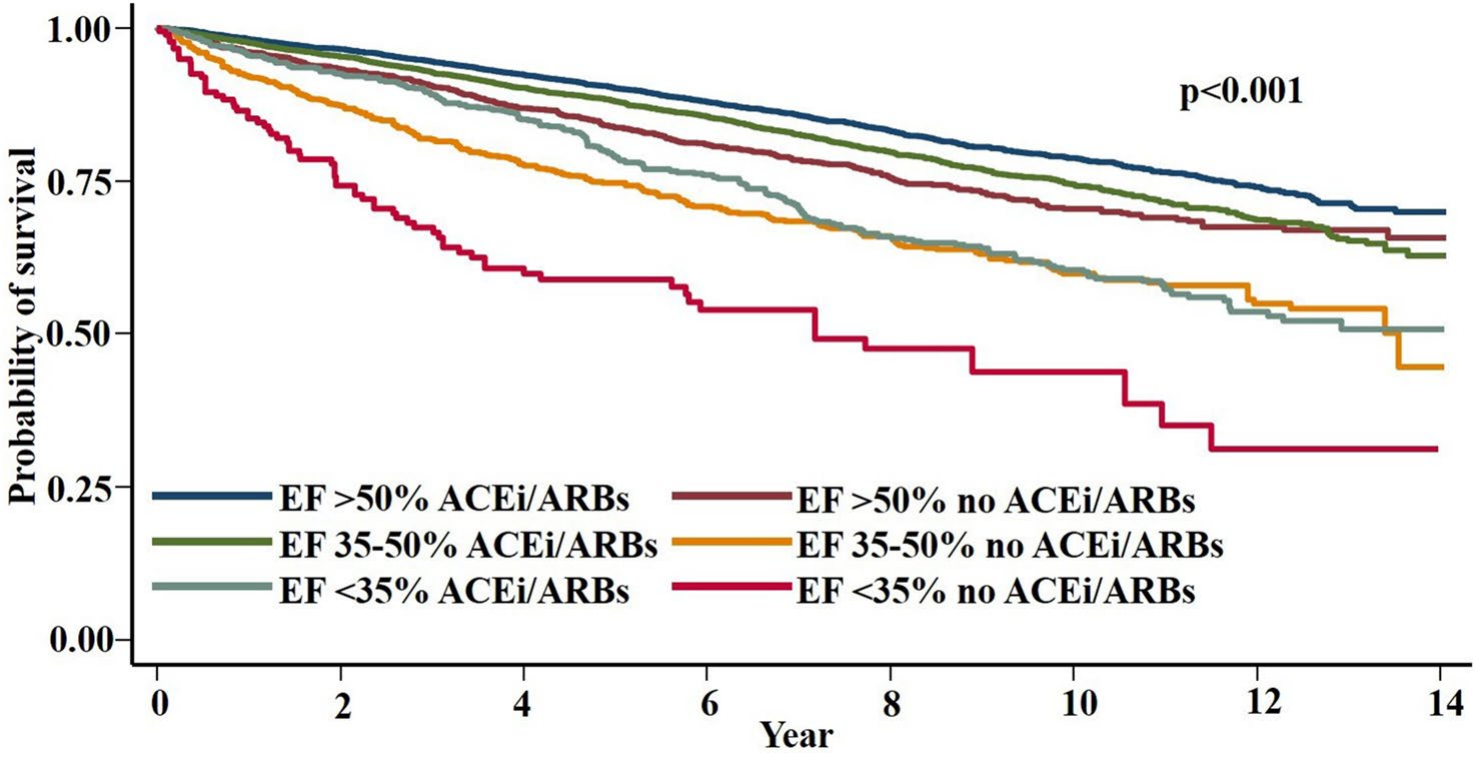
Kaplan–Meier survival analysis for all-cause mortality stratified by left ventricular ejection fraction. Within each group, ACEi/ARB therapy was associated with significantly lower mortality compared to no ACEi/ARB therapy (all *p* < 0.001). *ACEi* angiotensin converting enzyme inhibitor, *ARB* angiotensin receptor blocker, *EF* ejection fraction

On Kaplan–Meier analysis of the entire cohort, ACEi/ARB therapy was found to be associated with significantly reduced mortality (15.0% vs. 22.7%, *p* < 0.001; mean follow up 5.58 years; Fig. 3). This significant reduction in mortality was also present across each EF subgroup, with ACEi/ARB being associated with improved survival by 4.9%, 11.6% and 13.1% in patients with LVEF > 50%, 35–50% and < 35% respectively, suggesting a trend of increased therapeutic benefit with increasing LV dysfunction (Fig. 3).

**Fig. 4:**
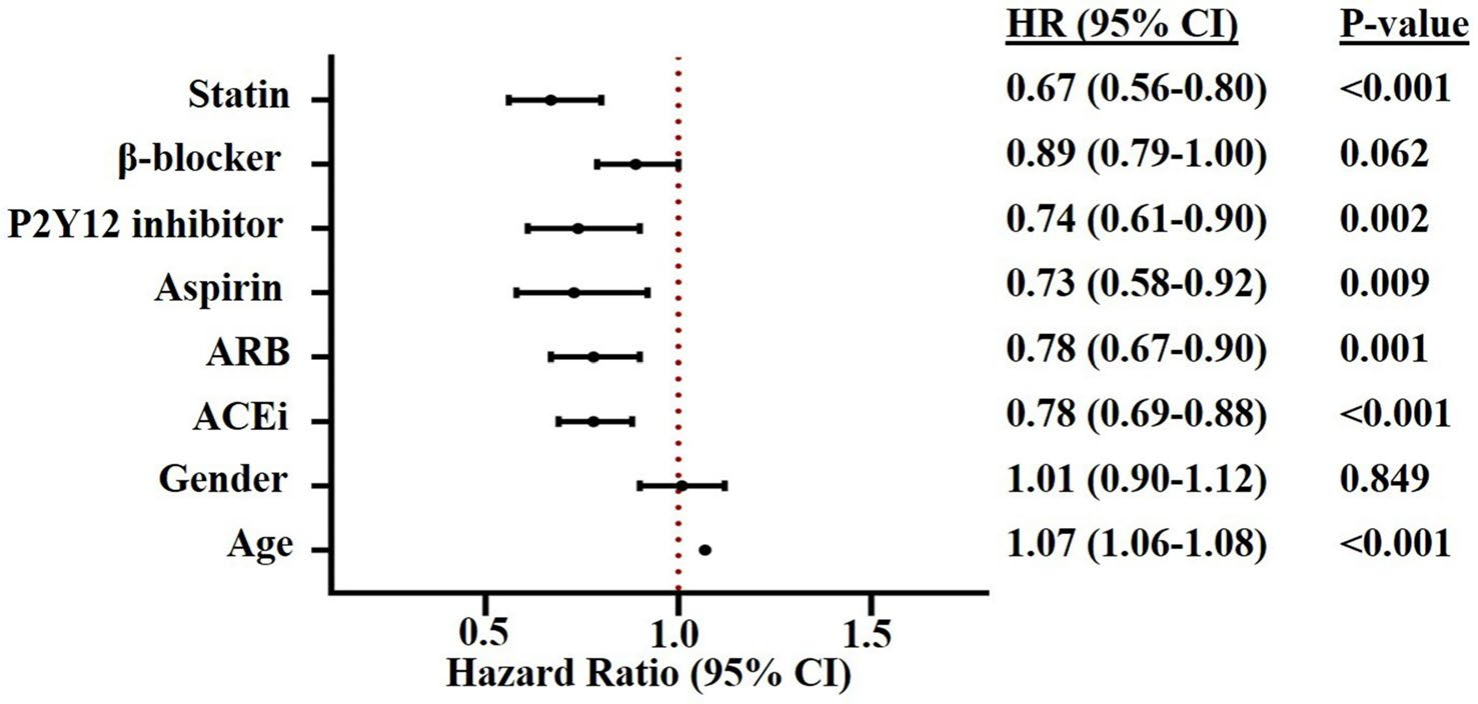
Cox proportional hazards survival analysis for all-cause mortality of the entire cohort (*N* = 21,388). Value of < 1.0 indicates improved survival. *ACEi* angiotensin converting enzyme inhibitor, *ARB* angiotensin receptor blocker

For the unselected cohort, Cox proportional hazards modelling demonstrated that ACEi and ARB use were independently associated with reduced mortality (Fig. 4, Supplementary Table 1). On subgroup analysis with patients stratified by EF%, ACEi/ARB remained independently associated with improved survival in those with EF 35–50 and > 50% (both *p* < 0.001); however, did not reach significance in those with EF < 35% despite a trend towards survival benefit (HR 0.69, CI 0.45–1.1, *p* = 0.088; Supplementary Table 1). A full table of all variables analyzed by Cox proportional hazards modelling can be found in Supplementary Table 1.

## Discussion

The key findings of this study were that (1) the use of ACEi/ARB post PCI for NSTEMI/STEMI is high (83.8%); (2) ACEi/ARB therapy in patients post-PCI for NSTEMI or STEMI is associated with a significant long-term survival benefit; (3) this survival benefit is evident in patients with both preserved and reduced LV function.

Current international guidelines recommend the use of ACEi (or ARBs when ACEi are not tolerated) for patients with STEMI and NSTEMI with concomitant heart failure, LAD lesion, diabetes, or hypertension, and in those with stable CKD after NSTEMI with Class IA evidence [4–8]. Lower levels of evidence support their use outside of these criteria [4–8]. The current study found 83.8% of patients were prescribed ACEi/ARB therapy at 30 days post PCI. Given that 60.8% had hypertension, 43.9% had an EF < 50%, 21.1% were diabetic, 20.4% had an eGFR < 60, and 4.2% had reported heart failure at baseline prior to PCI; while 35.5% of presentations involved the left anterior descending or left main coronary arteries, prescribing practices appear to follow current guidelines. Use of ACEi/ARB also appeared to be associated with the severity of the presentation; with ACEi/ARB use significantly greater in those who had a STEMI compared to those with a NSTEMI or had an out-of-hospital cardiac arrest. The greater proportion of patients on ACEi/ARB with STEMI compared to NSTEMI is consistent with the greater level of evidence recommending their use in patients with STEMI (regardless of LV function) as per ACC/AHA guidelines. Notably, this evidence was obtained during the pre-PCI era [4, 8]. Recent evidence supports this prescribing practice in which RAS inhibition provided greater survival benefit in 6,762 pairs of unselected and propensity score-matched Korean patients with STEMI vs. NSTEMI patients 2-years post-PCI in an observational study [12].

The present study found that ACEi/ARB therapy was associated with significantly reduced long-term all-cause mortality in patients post-PCI for MI by Kaplan–Meier and Cox proportional hazards analyses of the entire cohort. This is consistent with original reports undertaken prior to PCI becoming standard practice. More recent smaller cohort studies have provided data in the current PCI era, where ACEi/ARB therapy was associated with significantly reduced 4-year all-cause mortality in 813 unselected and propensity-matched pairs of patients post-PCI for NSTEMI in Spain [13]; and was associated with significantly improved 5-year survival in 5563 unselected and propensity-matched Japanese patients presenting with MI (with > 80% of patients treated with PCI) [14].

There is currently less direct evidence supporting ACEi/ARB in patients with preserved LV function following PCI for a MI. Thus, the current study stratified patients by EF% (EF < 35%, 35–50% and > 50%) and revealed that ACEi/ARB therapy was associated with significantly higher survival across all subgroups irrespective of LVEF on Kaplan–Meier analysis. This provides important contemporary evidence to support ACEi/ARB therapy use in all patients post-PCI for STEMI/NSTEMI, regardless of LV function, with particular relevance to the modern era of early revascularization with primary PCI. Cox proportional hazards modelling demonstrated that ACEi/ARB therapy was independently associated with improved survival for those with EF 35–50 and > 50%, but despite a clear trend, did not reach significance in those with EF < 35%, likely attributable to the smaller sample size in this subgroup.

Analysis of ACEi and ARB therapy by Cox proportional modelling revealed each to be associated with a statistically significant survival benefit for the entire unselected cohort. Strong evidence exists for ACEi use in reducing mortality in LV dysfunction post MI in the pre-PCI era [15–17], while evidence for the use of ACEi in those with preserved LV function post MI has been an area of debate. HOPE and EUROPA placebo-controlled trials in patients with stable CAD and without heart failure reported ACEi to significantly reduce mortality; however only 52% and 64% of patients had a prior MI, and just 18% and 55% had undergone revascularization in each trial, respectively [18, 19]. In contrast, the placebo-controlled PEACE trial found the ACEi Trandolapril to have no significant survival benefit in patients with stable CAD without heart failure, but again contained low rates of prior MI (55%) and PCI (42%) [20]. Trials focused on investigating the effect of ACEi/ARB in secondary prevention with preserved LV function are scarce, with one placebo-controlled study of 406 Japanese patients with a history of coronary intervention and preserved LVEF reporting Candesartan significantly reduced cardiovascular death (RR 0.47; 95% CI 0.24–0.93, *p* = 0.03) [21]. In contrast, a more recent study of 988 patients with STEMI and preserved EF that underwent PCI showed no long-term survival benefit of ACEi/ARB therapy after a median of 4.6 years (HR 0.86, CI 0.56–1.33, *p* = 0.50) [22]. The reasons why that study conflicts with the current findings may be due to the much smaller sample size, and exclusion of patients with eGFR < 60 mL/min/1.73 m^2^ or NSTEMI. Furthermore, differences in study period (2002–2011 vs. 2005–2018) may also account for the variation in results due to changes in PCI practices. Recent prospective cohort and observational studies report ACEi and ARB therapy were each associated with significant and comparable survival benefits in patients presenting with STEMI and NSTEMI that underwent PCI in patients with preserved LV function [23, 24]. Taken together, both the current and recent relevant studies support the use of ARBs/ACEi in patients post PCI with preserved EF.

The efficacy of ACEi–ARB in modifying outcomes after PCI rely upon tolerance to these medications. The most common causes of intolerance include drug reactions, hypotension or hyperkalaemia often associated with renal impairment. Consistent with this, in the current study, those not-receiving ACEi/ARB therapy had a significantly reduced eGFR compared to those receiving ACEi/ARB therapy. Having a reduced renal function may potentially carry a worse prognosis for these patients and theoretically may have exaggerated the apparent association of ACEi/ARB therapy with improved long-term survival.

Observing patient medications, a significantly greater proportion of patients in the ACEi/ARB group were on aspirin, statin, beta-blockers and P2Y12 inhibitors; all of which are individually reported to improve survival after MI [4, 6–8]. The reason for this imbalance is unclear. It could be hypothesized that patients not able to tolerate ACEi/ARB due to hypotension may also not have been able to tolerate beta-blockers. Interestingly, on Cox proportional hazards modelling, beta-blockers were not associated with significantly improved survival in this study. Patient characteristics and post-PCI status do not provide clear evidence why statin and anti-platelet therapies were significantly less used in patients not on ACEi/ARBs. The most common contraindication to antiplatelet therapy is bleeding risk, namely gastrointestinal and intracranial, unfortunately neither of which were recorded in the current study. Lastly, a greater proportion of patients on ACEi/ARB therapy were taking a statin at 30 days despite the presence of dyslipidaemia being similar between the two groups. One contributing factor for this may be due to drug intolerances, suggested by the higher proportion of non-ACEi–ARB patients on ezetimibe. Despite all of these differences, ACEi/ARB therapy remained a statistically significant and independent predictor of mortality on Cox multivariable analysis.

## Limitations of study

There are several limitations in this study, namely its retrospective nature, possible selection bias, and unmeasured confounding factors which are inherent to all observational registry studies. However, these data have been collected prospectively since 2005. The findings comparing ACEi/ARB therapy to no ACEi/ARB therapy were based on medication use at 30-day follow-up, with the reason for cessation prior to this time-point not collected within our dataset. Unexpectedly, several medications used for secondary prevention after ACS were disproportionally used in the ACEi/ARB group as described above. The dose, specific drug type, long-term adherence or discontinuation of ACEi/ARB and/or other medications after 30 days were not recorded, although the latter would potentially diminish the associated survival benefits with ACEi/ARB. With the focus of this study on long-term survival outcomes, only patients that were alive and contactable at 30 days were included, thus any potential clinical effects of ACEi/ARB therapy prior to 30 days of PCI were not assessed, nor were major adverse cardiovascular events, change in LVEF, or other parameters of patient health recorded as part of this study. Lastly, routine use of Angiotensin Reception Neprilysin Inhibitors (ARNIs) in Australia was only commenced in the final year of our dataset and was therefore not included.

## Conclusions

This large observational study found ACEi/ARB therapy was associated with significant long-term survival benefit in patient’s post-PCI for STEMI/NSTEMI. This survival benefit is apparent in patients with both preserved and reduced LV function. These findings provide contemporary evidence to support the use of these agents in patients treated with PCI for STEMI/NSTEMI, irrespective of their baseline left ventricular function.

## Supplementary Information

Below is the link to the electronic supplementary material.

Supplementary file1 (DOCX 26 kb)
