# Supplementary material for: Role of renin–angiotensin system antagonists on long-term mortality post-percutaneous coronary intervention in reduced and preserved ejection fraction
Source: Clin Res Cardiol. 2022 Jan 20;111(7):776–86. doi: 10.1007/s00392-021-01985-x (PMC9242972; doi:10.1007/s00392-021-01985-x)
Supplement: Supplementary file 1 — Supplementary file1 (DOCX 26 kb) [file 392_2021_1985_MOESM1_ESM.docx]

**SUPPLEMENTAL TABLE 1.** **Cox proportional multivariate predictor of mortality hazard.**

|  | **Whole Cohort** | | | **LVEF<35%** | | | **LVEF 35-50%** | | | **LVEF >50%** | | |
| --- | --- | --- | --- | --- | --- | --- | --- | --- | --- | --- | --- | --- |
|  | **HR** | **95% CI** | **p value** | **HR** | **95% CI** | **p value** | **HR** | **95% CI** | **p value** | **HR** | **95% CI** | **p value** |
| Never smoked | 0.63 | 0.55-0.73 | <0.001 | 0.92 | 0.57-2.02 | 0.821 | 0.7 | 0.56-0.89 | 0.003 | 0.55 | 0.45-0.67 | <0.001 |
| Other rhythm | 0.65 | 0.48-0.88 | 0.005 | 0.6 | 0.23-1.54 | 0.292 | 0.71 | 0.23-1.54 | 0.292 | 0.56 | 0.36-0.88 | 0.012 |
| Statin | 0.67 | 0.56-0.80 | <0.001 | 0.28 | 0.16-0.48 | <0.001 | 0.62 | 0.46-0.83 | 0.001 | 0.85 | 0.65-1.10 | 0.206 |
| Aspirin | 0.73 | 0.58-0.92 | 0.009 | 0.88 | 0.41-1.91 | 0.764 | 0.67 | 0.48-0.93 | 0.016 | 0.77 | 0.54-1.13 | 0.189 |
| P2Y12 inhibitor | 0.74 | 0.61-0.90 | 0.002 | 0.57 | 0.30-1.09 | 0.09 | 0.84 | 0.63-1.13 | 0.249 | 0.73 | 0.54-0.97 | 0.033 |
| Sinus rhythm | 0.75 | 0.64-0.87 | <0.001 | 0.55 | 0.33-0.93 | 0.025 | 0.84 | 0.66-1.06 | 0.141 | 0.71 | 0.57-0.88 | 0.002 |
| ACEi/ARB | 0.76 | 0.67-0.85 | <0.001 | 0.69 | 0.45-1.1 | 0.088 | 0.72 | 0.60-0.86 | <0.001 | 0.81 | 0.69-0.95 | 0.01 |
| ACEi | 0.78 | 0.69-0.88 | <0.001 | 0.66 | 0.43-1.03 | 0.07 | 0.73 | 0.60-0.88 | 0.001 | 0.86 | 0.73-1.01 | 0.072 |
| ARB | 0.78 | 0.67-0.90 | 0.001 | 0.7 | 0.39-1.24 | 0.232 | 0.81 | 0.64-1.03 | 0.081 | 0.78 | 0.64-0.95 | 0.015 |
| Family history CVD | 0.82 | 0.74-0.91 | <0.001 | 1 | 0.67-1.48 | 0.991 | 0.86 | 0.73-0.99 | 0.05 | 0.78 | 0.68-0.90 | 0.001 |
| Dyslipidemia | 0.83 | 0.75-0.93 | 0.001 | 1.1 | 0.73-1.66 | 0.633 | 0.82 | 0.69-0.97 | 0.018 | 0.82 | 0.71-0.95 | 0.009 |
| Previous smoked | 0.84 | 0.74-0.96 | 0.011 | 0.85 | 1.08-2.61 | 0.021 | 0.87 | 0.70-1.08 | 0.203 | 0.77 | 0.64-0.92 | 0.004 |
| β-blocker | 0.89 | 0.79-1.00 | 0.062 | 0.66 | 0.38-1.14 | 0.142 | 0.85 | 0.70-1.03 | 0.093 | 0.95 | 0.81-1.11 | 0.522 |
| BMI | 0.98 | 0.97-0.99 | <0.001 | 0.95 | 0.91-0.99 | 0.009 | 0.99 | 0.97-1.01 | 0.255 | 0.98 | 0.96-0.99 | 0.001 |
| Female gender | 1.01 | 0.90-1.12 | 0.849 | 1.33 | 0.87-2.04 | 0.188 | 0.91 | 0.76-1.09 | 0.298 | 1.03 | 0.88-1.19 | 0.738 |
| Age | 1.07 | 1.06-1.08 | <0.001 | 1.04 | 1.02-1.06 | <0.001 | 1.07 | 1.06-1.08 | <0.001 | 1.08 | 1.07-1.09 | <0.001 |
| LVEF 35-50% | 1.11 | 1.00-1.22 | 0.048 |  |  |  |  |  |  |  |  |  |
| Cerebrovascular disease | 1.19 | 1.02-1.39 | 0.022 | 0.93 | 0.49-1.77 | 0.832 | 1.3 | 1.0-1.66 | 0.038 | 1.11 | 0.90-1.37 | 0.343 |
| Previous MI | 1.22 | 1.08-1.37 | 0.001 | 1.75 | 1.12-2.73 | 0.013 | 1.13 | 0.93-1.36 | 0.217 | 1.27 | 1.07-1.50 | 0.006 |
| Previous CABG | 1.27 | 1.08-1.49 | 0.003 | 1.18 | 0.68-2.05 | 0.546 | 1.27 | 0.99-1.64 | 0.06 | 1.25 | 0.99-1.58 | 0.06 |
| NIDDM | 1.31 | 1.16-1.48 | <0.001 | 1.68 | 1.08-2.61 | 0.021 | 1.34 | 1.10-1.62 | 0.003 | 1.31 | 1.11-1.56 | 0.001 |
| Heart Failure | 1.31 | 1.09-1.56 | 0.003 | 1.17 | 0.73-1.87 | 0.498 | 1.31 | 0.99-1.73 | 0.05 | 1.53 | 1.15-2.03 | 0.003 |
| Obstructive sleep apnea | 1.37 | 1.10-1.72 | 0.005 | 2.01 | 0.79-5.06 | 0.137 | 1.13 | 0.78-1.64 | 0.504 | 1.47 | 1.09-1.98 | 0.013 |
| eGFR 30-60 | 1.37 | 1.23-1.53 | <0.001 | 1.36 | 0.91-2.02 | 0.126 | 1.32 | 1.11-1.57 | 0.002 | 1.45 | 1.24-1.69 | <0.001 |
| Prior valve surgery | 1.39 | 0.98-1.99 | 0.067 | 0.63 | 0.15-2.64 | 0.531 | 1.91 | 1.11-3.29 | 0.02 | 1.19 | 0.71-2.01 | 0.512 |
| PVD | 1.39 | 1.20-1.61 | <0.001 | 0.98 | 0.54-1.77 | 0.545 | 1.39 | 1.11-1.75 | 0.004 | 1.51 | 1.23-1.86 | <0.001 |
| Chronic lung disease | 1.51 | 1.33-1.70 | <0.001 | 1.56 | 1.02-2.41 | 0.039 | 1.51 | 1.24-1.83 | <0.001 | 1.64 | 1.38-1.95 | <0.001 |
| LVEF <35% | 1.53 | 1.28-1.83 | <0.001 |  |  |  |  |  |  |  |  |  |
| Rheumatoid arthritis | 1.62 | 1.28-2.05 | <0.001 | 1.35 | 0.52-3.51 | 0.533 | 1.57 | 1.07-2.31 | 0.022 | 1.61 | 1.16-2.23 | 0.004 |
| Spironolactone | 1.7 | 1.38-2.08 | <0.001 | 2.12 | 1.37-3.25 | 0.001 | 1.85 | 1.35-2.53 | <0.001 | 1.32 | 0.87-1.99 | 0.19 |
| IDDM | 1.73 | 1.47-2.05 | <0.001 | 1.07 | 0.57-2.02 | 0.821 | 1.95 | 1.52-2.51 | <0.001 | 1.91 | 1.49-2.45 | <0.001 |
| eGFR <30 | 3.35 | 2.81-3.99 | <0.001 | 4.84 | 2.72-8.59 | <0.001 | 2.62 | 1.98-3.48 | <0.001 | 3.86 | 2.97-5.01 | <0.001 |

ACEi, angiotensin converting enzyme inhibitor; ARB angiotensin II receptor blocker; BMI, body mass index (kg/m^2^); CABG, coronary artery bypass graft; CVD, cardiovascular disease; eGFR, estimated glomerular filtration rate (ml/min/1.73m^2^); IDDM, insulin-dependent diabetes mellitus; LVEF, left ventricular ejection fraction; MI, myocardial infarction; NIDDM, non-IDDM; PVD, peripheral vascular disease. NIDDM and IDDM were compared against ‘no diabetes’; smoking was compared against ‘current smoker’; heart rhythms were compared with ‘atrial fibrillation’; eGFR was compared to eGFR >60; LVEF’s were compared to >50%.
